# Evidence for reduced lymphatic CSF absorption in the H-Tx rat hydrocephalus model

**DOI:** 10.1186/1743-8454-5-15

**Published:** 2008-10-16

**Authors:** Matthias Rammling, Meenu Madan, Leena Paul, Babak Behnam, Jogi V Pattisapu

**Affiliations:** 1 Hydrocephalus Research Laboratory, Burnett School of Biomedical Sciences, College of Medicine, University of Central Florida, Orlando, FL 32816, USA

## Abstract

**Background:**

There is mounting evidence that spinal fluid absorption takes place not only at the arachnoid villi, but also at several extracranial sites, which might serve as a reserve mechanism for, or be primarily involved in the absorption of CSF in hydrocephalus.

**Methods:**

We compared the nasal lymphatic pathway in congenital Hydrocephalus-Texas (H-Tx) rats in unaffected and affected hydrocephalic (HC) siblings with that of control Sprague Dawley (SD) rat pups. The animals were examined after immediate *post mortem *injection of Evan's blue dye into the cisterna magna at 6 and 10 days of age. The specimens were evaluated for amount of dye penetration into the nasal passages.

**Results:**

We found more dye visualization in the olfactory regions of control SD (14/16 at P6, 14/16 at P10) and unaffected H-Tx (13/17 at P6, 13/16 at P10) compared with HC animals (0/14 at P6, 3/15 at P10). This difference was more pronounced at 10 days of age. The dye was not visualized in the cervical lymph nodes or venous channels in these acute experiments.

**Conclusion:**

The results of this study suggest that nasal lymphatic cerebrospinal fluid absorption is reduced in the H-Tx rat hydrocephalus model.

## Background

Hydrocephalus is a lifelong condition of cerebrospinal fluid (CSF) imbalance, leading to seizures, neurological and cognitive dysfunction, or death if left untreated. The condition is not a single pathologic entity, nor is it a simple, well-defined disease process; rather, it encompasses a diverse group of clinical situations sharing a common feature of increased intracranial pressure (ICP) resulting from an imbalance of CSF secretion and absorption. A better understanding of these as yet undiagnosed mechanisms may offer therapeutic options for rebalancing CSF dynamics before fluid accumulation occurs.

We must reconsider the conventional wisdom that primary CSF absorption occurs through the arachnoid granulations, since there is limited evidence concerning fibrosis or anatomic obstruction at these sites in hydrocephalus [1-4]. Yet there is increasing evidence for communication between the CSF pathways and the extracranial lymphatic system, mostly by the nasal lymphatics or the optic nerve sheath into the facial venous system, elegantly described by Johnston *et al *to be functional in most mammals, including humans [5-9]. This group found that approximately 50% of CSF is cleared from the cranial compartment by the nasal lymphatic vessels, which play a major role in bulk flow of spinal fluid into the venous system. Other studies confirm that CSF drains along cranial nerves eventually into the lymphatic channels [10-13]. Accumulation of excessive spinal fluid, especially under increased pressure, could implicate a problem in these mechanisms. [14-19].

We hypothesize that obstruction of CSF flow via the cribriform plate into the facial lymphatic pathways is related to hydrocephalus in the Hydrocephalus-Texas (H-Tx) rat model. We investigated nasal lymphatic pathways in hydrocephalus by injecting Evan's blue dye into the cisterna magna of Sprague Dawley (SD) rats, unaffected H-Tx rats, and their affected hydrocephalic (HC) siblings at post natal days P6 and P10, and studied the amount of dye penetration in the nasal regions.

## Methods

### Animals

The H-Tx rat was initially identified in 1981 by Kohn, *et al*. [20], and a colony from animals originally from the University of Florida is maintained at our institution by brother-sister mating. Although the genetic cause of the H-Tx mutation is unknown, it is a widely recognized animal model that mirrors the human condition. Hydrocephalus occurs in approximately 20–40% of each litter, with obstruction of the aqueduct on day E18 [21,22]. The ventricular system dilates rapidly, as evidenced by an enlarged, domed dorsal cranium evident by 1–2 days of age [20]. Most hydrocephalic rats die at 4–5 weeks of age if not treated by shunting procedures, which confer a normal life span [23,24].

The Sprague Dawley rats were purchased from Charles River Laboratories (Cary, North Carolina). All experiments were performed under the guidelines of the Animal Care Committee at the University of Central Florida. The animals were housed under 12-hr day-night cycles and had free access to food and water. A total of 112 rat pups were used for this study (Table 1).

**Table 1:** Evan's blue dye penetration into olfactory turbinates of control and hydrocephalic rats.

	**Post-mortem injection**			**Live injection**		
	***SD***	***H-TX***	***HC***	***SD***	***H-TX***	***HC***
P6	14/16	13/17	0/14	---	---	---
P10	14/16	13/16	3/15	6/6	6/6	0/6

Numbers of postnatal rats with Evan's blue in the olfactory turbinates out of the total number of animals used. SD: Sprague Dawley, H-Tx: unaffected H-Tx, HC: hydrocephalic H-TX.

### Magnetic resonance imaging

**Figure 1:**
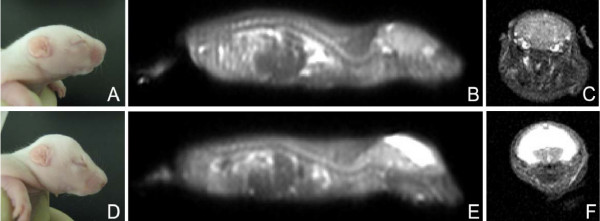
**Photographs and MR scans of unaffected H-Tx and hydrocephalic H-Tx rat pups at P10**. Phenotype of H-Tx pups at P10: A, unaffected H-Tx pup and D, hydrocephalic pup. Note the enlarged domed head, usually evident by 3 days of age in D. Representative sagittal MR scans of unaffected H-Tx (B) and HC animal (E) showing enlargement of lateral ventricles, and normal sized cerebellum. Coronal T2-weighted slices confirm small lateral ventricles of unaffected H-Tx (C) and the large lateral ventricles in the HC pup (F).

Ten-day old rat pups were anesthetized with isofluorane and immobilized in plastic tube restraining devices, T2-weighted 1 mm sagittal and coronal MR images were obtained using a Siemens Somatom 1.5 T magnet (Siemens, Berlin, Germany, Figure 1).

### Tracer injection and visualization

In order to compare experiments using anesthetized animals with those after CO_2 _euthanasia, initial experiments were performed using 10-day old pups (n = 18) anesthetized using pentobarbital (20 mg/kg, i p). Evan's blue dye (50 μL, 2% in PBS) was injected into the cisterna magna subarachnoid space. The animals were sacrificed after 20 min by an additional injection of pentobarbital (50 mg/kg).

Ninety four animals (47 at P6 and 47 at P10) were sacrificed by CO_2 _inhalation and underwent immediate *post mortem *injection (30 μL at P6, and 50 μL at P10) of 2% Evan's blue dye in PBS into the cisterna magna subarachnoid space.

After dye injection, pups were kept in head-dependent supine position at -20°C for 24 h prior to sectioning. Sections 1 mm thick were made by hand in coronal or sagittal planes on dry ice, and images at 40× magnification were obtained under a dissecting microscope (Zeiss, STEMI SV 11, Germany), and captured by a digital camera. These images were used to examine for dye penetration from the cribriform plate into the olfactory turbinates and nasal pathways. Alpha Imager 2200 software was used to quantify the amount of dye visualized in the nasal regions between the 3 groups of animals (Alpha Innotech Corp. CA). We arbitrarily assigned the average value of HC animals as reference (baseline of 1), and compared the SD and H-Tx groups against this value for relative dye intensity.

## Results

**Figure 2:**
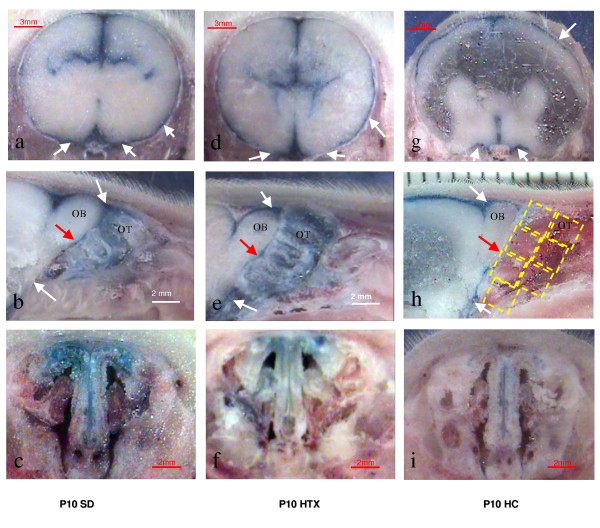
**Surface view of the frozen whole head showing distribution of 50 μl of 2% Evan's blue dye injected into the cistern magna of rats *post mortem *at 10 days of age. Note blue dye in the subarachnoid spaces over the cortex and the skull base in all 3 groups (white arrows)**. Top row: coronal views at level of lateral ventricles (a, d, g). Note enlarged lateral ventricles in hydrocephalic animal (g). Middle row: Olfactory region in the sagittal plane of SD (b), H-Tx (e) and affected hydrocephalic animals (h). The overlay grid indicates region of olfactory turbinates for semi-quantitative analysis between groups. Note the dye is present in the subarachnoid spaces around the olfactory bulb (OB) in all animals. The blue tracer passed through the cribriform plate (red arrows) into the olfactory turbinates (OT) of the SD (b) and H-Tx (e) pups. Less dye was observed in the olfactory turbinates of hydrocephalic animals (h). Bottom row: coronal sections showing the nasal regions, showing less dye in the hydrocephalic animal (i). White arrows indicate Evan's blue in subarachnoid spaces, red arrows identify cribriform plate. Reference scales are provided as a ruler (mm) or as a longitudinal bar.

The hydrocephalic pups were phenotypically different with their characteristic domed shaped head (Figure 1), decreased spontaneous activity, and limb stiffness. Ventricular dilatation, which was confirmed by MR imaging (Figure 1) and at sectioning, was more pronounced at the older age (P10). In all groups, the Evan's blue dye was visualized in the subarachnoid spaces over the cerebral convexities and at the skull base (Figure 2a, d, g). The Evan's blue dye was visualized in the subarachnoid spaces, penetrating the nasal passages in the unaffected H-Tx and SD control animals, and less evident in the hydrocephalic rat pups.

Eighteen animals at P10 were initially utilized for live injection of Evan's blue dye under pentobarbital sedation prior to sacrifice. Adequate visualization of dye within the olfactory turbinates was seen in the six SD and six unaffected H-Tx pups, but no visible dye was noted in the six affected HC animals.

The larger series with 47 pups at P6 and 47 at P10 was performed using *post mortem *injection after CO_2 _euthanasia. Evan's blue dye was visible within the nasal passages of 14/16 pups at P6 and P10 in the SD animals. Similarly, in the unaffected H-Tx group, dye was visualized in 13/17 of pups at P6 and 13/16 at P10. However, there was no dye visible in the olfactory turbinates of 14 HC animals at P6 and only 3/15 had visible dye in the olfactory regions at P10 (Table 1, Figure 2). The dye was not visualized in the cervical lymph nodes or venous channels in these acute experiments.

**Figure 3:**
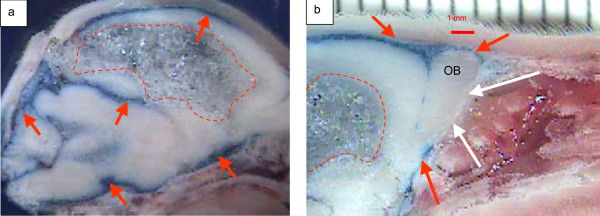
**Dye distribution in subarachnoid space of hydrocephalic animals after immediate *post-mortem *injection of 2% Evan's blue dye into the cisterna magna. Anterior is to the right in each picture**. a) Parasagittal view of P6 hydrocephalic rat showing dye posteriorly in subarachnoid spaces (arrows), with large lateral ventricle (dashed lines). b) Higher magnification of frontal area in at P10, showing lack of dye penetration into the nasal passages. Note dye (red arrows) at skull base anterior and posterior to the olfactory bulb (OB) (white arrows). Also visible is the large ventricle containing frozen CSF (dashed lines). (1 mm marker above). Red arrows – Evan's blue dye in subarachnoid spaces. White arrows – olfactory bulb (OB).

The dye visualized in the hydrocephalic animals was arbitrarily assigned a baseline reference value of 1 (Figure 2h). We then compared the relative dye intensity in the other 2 groups (unaffected H-Tx and control SD animals) against this background value, to evaluate the relative expression of blue dye within the nasal sinuses and olfactory turbinates among the three subgroups (Table 2). Semi-quantitative analysis of the blue dye between the three groups at P6, identified an over 3-fold increase in dye concentration in the SD control and unaffected H-Tx siblings as compared with the hydrocephalic pups. This difference was more pronounced at the older age (P10), where the dye concentration was 5.7 times the HC values in the SD animals and 5 times greater in the unaffected H-Tx siblings (Figure 3).

**Table 2:** Relative fold increase in Evan's blue dye concentration into the olfactory turbinates of control rats compared to hydrocephalic rats.

**Age**	**SD**	**H-TX**	**HC**
**P6**	3.3	3.4	1*
**P10**	5.7	5.0	1*

Evan's blue dye penetration of the olfactory turbinates was determined by an overlay grid (in the sagittal plane) and colorimetric values were analyzed. The dye concentration visualized in the HC animals was arbitrarily assigned a baseline reference value of 1(*). We compared the relative amount of dye visualized in the H-Tx and SD animals to this reference value. These two groups had several fold increase of dye concentration in turbinates compared to HC animals.

## Discussion

Initial concepts of hydrocephalus pathophysiology were based on a limited understanding of CSF dynamics, alternative flow pathways, and the compensatory mechanisms involved. While ongoing fundamental studies explore the complex mechanisms of normal brain development, those involved in the molecular basis of hydrocephalus remain unexplained. Thus, medical management of this lifelong condition remains elusive, with few research efforts in this direction.

Anatomical and tracer studies have identified CSF flow in the subarachnoid spaces surrounding the olfactory nerves into the cribriform plate into a network of lymphatic ducts in the olfactory turbinates [25-27]. The fluid is then conveyed into progressively larger lymph channels and ultimately deposited into the venous system in the cervical region [16,17,28]. In rodents, these associations are not observed until four days after birth, when CSF production significantly increases. Timing of this occurrence perhaps suggests a pressure-dependent mechanism to open and maintain these CSF outflow channels [10].

Recent studies by Nagra, *et al*. identified decreased flow in the nasal passages of rats with acquired hydrocephalus by kaolin injection, where intraventricular injection of Evan's blue dye did not penetrate the subarachnoid spaces and was not visualized in the nasal passages [29]. Scarring or obliteration of the arachnoid spaces near the olfactory region may contribute to the diminished flow via these pathways in this induced hydrocephalus model. Evan's blue dye was used for its ease of handling, and the ability to assess the anatomic CSF localization in the *post mortem *animals [10]. Evan's blue dye in PBS was found to be very effective in delineating the pathways and appears to be as effective as dye linked to protein (Johnston, M, personal communication).

In our animals, dye penetration was limited in the nasal passages, despite adequate representation of dye within the subarachnoid spaces in the basal cisterns and surrounding the cribriform plate (Figure 3). We therefore postulate that CSF in the hydrocephalic animals had access to the cribriform plate and the nasal passage for egress, suggesting a primary obstruction at these sites. Studies to identify further delineate the anatomic sites of blockage are necessary.

Although these nasal pathways for CSF absorption are known to exist in several species, this is the first study to document their connection with congenital hydrocephalus. Since hydrocephalus is a multifactorial condition, perhaps diminished nasal lymphatic outflow tracts increase the propensity to develop the condition. There might be a triggering factor which cannot be overcome in a certain number of animals leading to increased susceptibility to a stimulus such as infection or subarachnoid hemorrhage.

In animal experiments, obstruction of the cribriform plate reduces CSF clearance and leads to increased ICP, but it is unclear if it is involved in hydrocephalus [8,15,30]. Ludemann, *et al. *studied the ultra structure of CSF outflow along the optic nerve and identified pore-like openings in a thin neurothelial layer extending into the lymphatics, which might respond to elevated CSF pressure [31]. Similar mechanisms might play a role in increased intracranial pressure in mammals.

In H-Tx animals the hydrocephalus is associated with aqueductal stenosis at E18, and the condition progressively worsens until death at 5–6 weeks of age [21]. However, the nasal pathways for CSF absorption develop at post natal day 4 in rats, which calls into question the possibility of a causal relationship between CSF drainage pathways and hydrocephalus [10]. It is plausible that the nasal lymphatic pathway is an outflow track dependent on pressure-driven mechanisms for proper development and function. This submucosal CSF egress might serve as a compensatory mechanism that fails when most needed. We studied the H-Tx animals at 6 days of age and later dates to assess the possibility of secondary, pressure-driven recruitment of these pathways. Dye penetration was more pronounced in normal animals at P10, and it was better defined in deeper ethmoid sinus regions of the older animals, suggesting some recruitment of these pathways over time. Evan's blue was not visualized in the cervical lymph nodes in these animals.

The nasal passages may constitute another route for CSF absorption that can be recruited either temporarily or permanently at times of increased intracranial pressure (such as hydrocephalus). Drugs administered intranasally may gain access to the brain via axonal transport along the olfactory receptor cells extending through the cribriform plate to the olfactory bulb [32]. We hypothesize that pore-like openings responsible for extracranial olfactory CSF drainage are obstructed in the affected (hydrocephalic) H-Tx animal, and they may be reopened using newer therapeutic measures.

## Conclusion

Our data suggest that the nasal lymphatic drainage pathway of CSF absorption is present in normal SD and unaffected H-Tx rat pups at 6 and 10 days of age and diminished or absent in hydrocephalic rat pups. The nasal lymphatic pathway is affected in rodents with congenital hydrocephalus, and may very well present a target for novel therapeutic agents in hydrocephalus. Future studies along these lines are warranted.

## Competing interests

The authors declare that they have no competing interests.

## Authors' contributions

MR carried out the experiment, data acquisition and interpretation, and drafting the manuscript. MM Alpha Imager data acquisition and interpretation, manuscript revision. LP Data acquisition and manuscript revision. BB Data acquisition and interpretation. JVP Project conception and design, revision of experiments, drafting manuscript. All authors read and approved the final manuscript.

## References

[B1] Massicotte EM, Del Bigio MR (1999). Human arachnoid villi response to subarachnoid hemorrhage: possible relationship to chronic hydrocephalus. J Neurosurg.

[B2] Mawera G, Asala SA (1996). The function of arachnoid villi/granulations revisited. Cent Afr J Med.

[B3] Johanson CE, Duncan JA, Klinge PM, Brinker T, Stopa EG, Silverberg GD (2008). Multiplicity of cerebrospinal fluid functions: New challenges in health and disease. Cerebrospinal Fluid Res.

[B4] Rennels ML, Gregory TF, Blaumanis OR, Fujimoto K, Grady PA (1985). Evidence for a 'paravascular' fluid circulation in the mammalian central nervous system, provided by the rapid distribution of tracer protein throughout the brain from the subarachnoid space. Brain Res.

[B5] Földi M, Gellert A, Kozma M, Poberai M, Zoltan O, Csanda E (1966). New contributions to the anatomical connections of the brain and the lymphatic system. Acta Anat (Basel).

[B6] Brinker T, Ludemann W, Berens von Rautenfeld D, Samii M (1997). Dynamic properties of lymphatic pathways for the absorption of cerebrospinal fluid. Acta Neuropathol.

[B7] Johnston M, Papaiconomou C (2002). Cerebrospinal fluid transport: a lymphatic perspective. News Physiol Sci.

[B8] Johnston M, Zakharov A, Papaiconomou C, Salmasi G, Armstrong D (2004). Evidence of connections between cerebrospinal fluid and nasal lymphatic vessels in humans, non-human primates and other mammalian species. Cerebrospinal Fluid Res.

[B9] Koh L, Zakharov A, Johnston M (2005). Integration of the subarachnoid space and lymphatics: is it time to embrace a new concept of cerebrospinal fluid absorption?. Cerebrospinal Fluid Res.

[B10] Koh L, Zakharov A, Nagra G, Armstrong D, Friendship R, Johnston M (2006). Development of cerebrospinal fluid absorption sites in the pig and rat: connections between the subarachnoid space and lymphatic vessels in the olfactory turbinates. Anat Embryol (Berl).

[B11] Caversaccio M, Peschel O, Arnold W (1996). The drainage of cerebrospinal fluid into the lymphatic system of the neck in humans. ORL J Otorhinolaryngol Relat Spec.

[B12] Lowhagen P, Johansson BB, Nordborg C (1994). The nasal route of cerebrospinal fluid drainage in man. A light-microscope study. Neuropathol Appl Neurobiol.

[B13] Weller RO, Kida S, Zhang ET (1992). Pathways of fluid drainage from the brain – morphological aspects and immunological significance in rat and man. Brain Pathol.

[B14] Boulton M, Flessner M, Armstrong D, Hay J, Johnston M (1998). Determination of volumetric cerebrospinal fluid absorption into extracranial lymphatics in sheep. Am J Physiol.

[B15] Mollanji R, Bozanovic-Sosic R, Silver I, Li B, Kim C, Midha R, Johnston M (2001). Intracranial pressure accommodation is impaired by blocking pathways leading to extracranial lymphatics. Am J Physiol Regul Integr Comp Physiol.

[B16] Bradbury MWB, Cserr HF, Johnston M (1985). Drainage of cerebral interstitial fluid and of cerebrospinal fluid into lymphatics. Experimental Biology of the Lymphatic Circulation.

[B17] Kida S, Pantazis A, Weller RO (1993). CSF drains directly from the subarachnoid space into nasal lymphatics in the rat. Anatomy, histology and immunological significance. Neuropathol Appl Neurobiol.

[B18] Boulton M, Flessner M, Armstrong D, Hay J, Johnston M (1997). Lymphatic drainage of the CNS: effects of lymphatic diversion/ligation on CSF protein transport to plasma. Am J Physiol.

[B19] Boulton M, Flessner M, Armstrong D, Mohamed R, Hay J, Johnston M (1999). Contribution of extracranial lymphatics and arachnoid villi to the clearance of a CSF tracer in the rat. Am J Physiol.

[B20] Kohn DF, Chinookoswong N, Chou SM (1981). A new model of congenital hydrocephalus in the rat. Acta Neuropathol.

[B21] Jones HC, Harris NG, Briggs RW, Williams SC (1995). Shunt treatment at two postnatal ages in hydrocephalic H-Tx rats quantified using MR imaging. Exp Neurol.

[B22] Pourghasem M, Mashayekhi F, Bannister CM, Miyan J (2001). Changes in the CSF fluid pathways in the developing rat fetus with early onset hydrocephalus. Eur J Pediatr Surg.

[B23] Miyazawa T, Sato K (1991). Learning disability and impairment of synaptogenesis in HTX-rats with arrested shunt-dependent hydrocephalus. Childs Nerv Syst.

[B24] Cai X, McGraw G, Pattisapu JV, von Kalm L, Willingham S, Socci D, Gibson JS (2000). Hydrocephalus in the H-Tx rat: a monogenic disease?. Exp Neurol.

[B25] Zakharov A, Papaiconomou C, Johnston M (2004). Lymphatic vessels gain access to cerebrospinal fluid through unique association with olfactory nerves. Lymphat Res Biol.

[B26] Nagra G, Koh L, Zakharov A, Armstrong D, Johnston M (2006). Quantification of cerebrospinal fluid transport across the cribriform plate into lymphatics in rats. Am J Physiol Regul Integr Comp Physiol.

[B27] Brodbelt A, Stoodley M (2007). CSF pathways: a review. Br J Neurosurg.

[B28] Zakharov A, Papaiconomou C, Djenic J, Midha R, Johnston M (2003). Lymphatic cerebrospinal fluid absorption pathways in neonatal sheep revealed by subarachnoid injection of Microfil. Neuropathol Appl Neurobiol.

[B29] Nagra G, Li J, McAllister JP, Miller J, Wagshul M, Johnston M (2008). Impaired lymphatic cerebrospinal fluid absorption in a rat model of kaolin-induced communicating hydrocephalus. Am J Physiol Regul Integr Comp Physiol.

[B30] Johanson CE, Duncan JA, Stopa EG, Baird A (2005). Enhanced prospects for drug delivery and brain targeting by the choroid plexus-CSF route. Pharm Res.

[B31] Ludemann W, Berens von Rautenfeld D, Samii M, Brinker T (2005). Ultrastructure of the cerebrospinal fluid outflow along the optic nerve into the lymphatic system. Childs Nerv Syst.

[B32] Graff CL, Pollack GM (2005). Nasal drug administration: potential for targeted central nervous system delivery. J Pharm Sci.

